# Supplementary material for: Functional organization of cytoplasmic inclusion bodies in cells infected by respiratory syncytial virus
Source: Nat Commun. 2017 Sep 15;8:563. doi: 10.1038/s41467-017-00655-9 (PMC5601476; doi:10.1038/s41467-017-00655-9)
Supplement: Supplementary file 1 — Supplementary Information [file 41467_2017_655_MOESM1_ESM.pdf]

Description of Supplementary Files

File Name: Supplementary Information
Description: Supplementary Figures and Supplementary Table.

File Name: Supplementary Movie 1
Description: **Movie of IBAGs dynamics.** Time-lapse microscopy of IBAGs in HEp-2 cells infected with RSV-M2-1mGFP. At 24h p.i., cells were imaged every 5 minutes in a chamber heated at 37°C, with a Leica SP8 confocal microscope. The resulting movies were visualized under the Imaris software. A representative movie out of 5 independent experiments is shown. The M2-1mGFP protein is visualized through its spontaneous green fluorescence.

File Name: Peer Review File

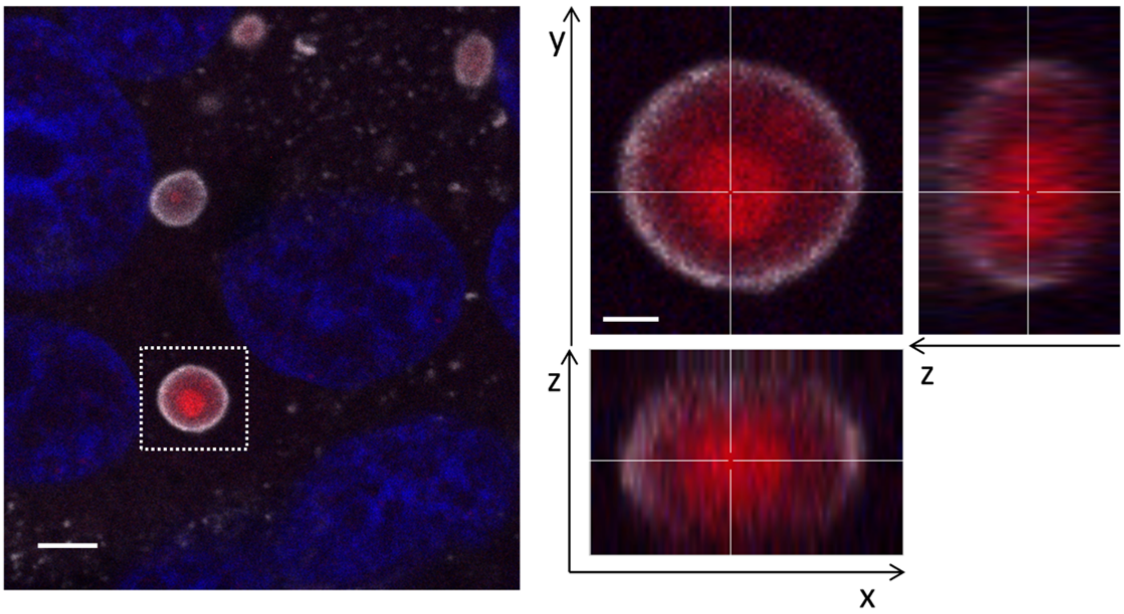

Supplementary Figure 1: 3D Localization of newly synthesized viral RNA in IBs of RSV infected cells
HEp-2 cells were treated as described as in Fig. 1 and images were taken under a Leica SP8 confocal microscope at 24h.p.i.. The 5EU incorporated in newly synthesized RNAs was detected using Alexa Fluor 647-azide (red) and cells were stained with an anti-N antibody (grey) and Hoechst 33258 (merge). Scale bar 5µm. The boxed area encloses IB that is shown magnified (zoom). Scale Bar 1µm. The 5EU distribution is depicted as a single X-Z section or a X-Z projection of the IB.

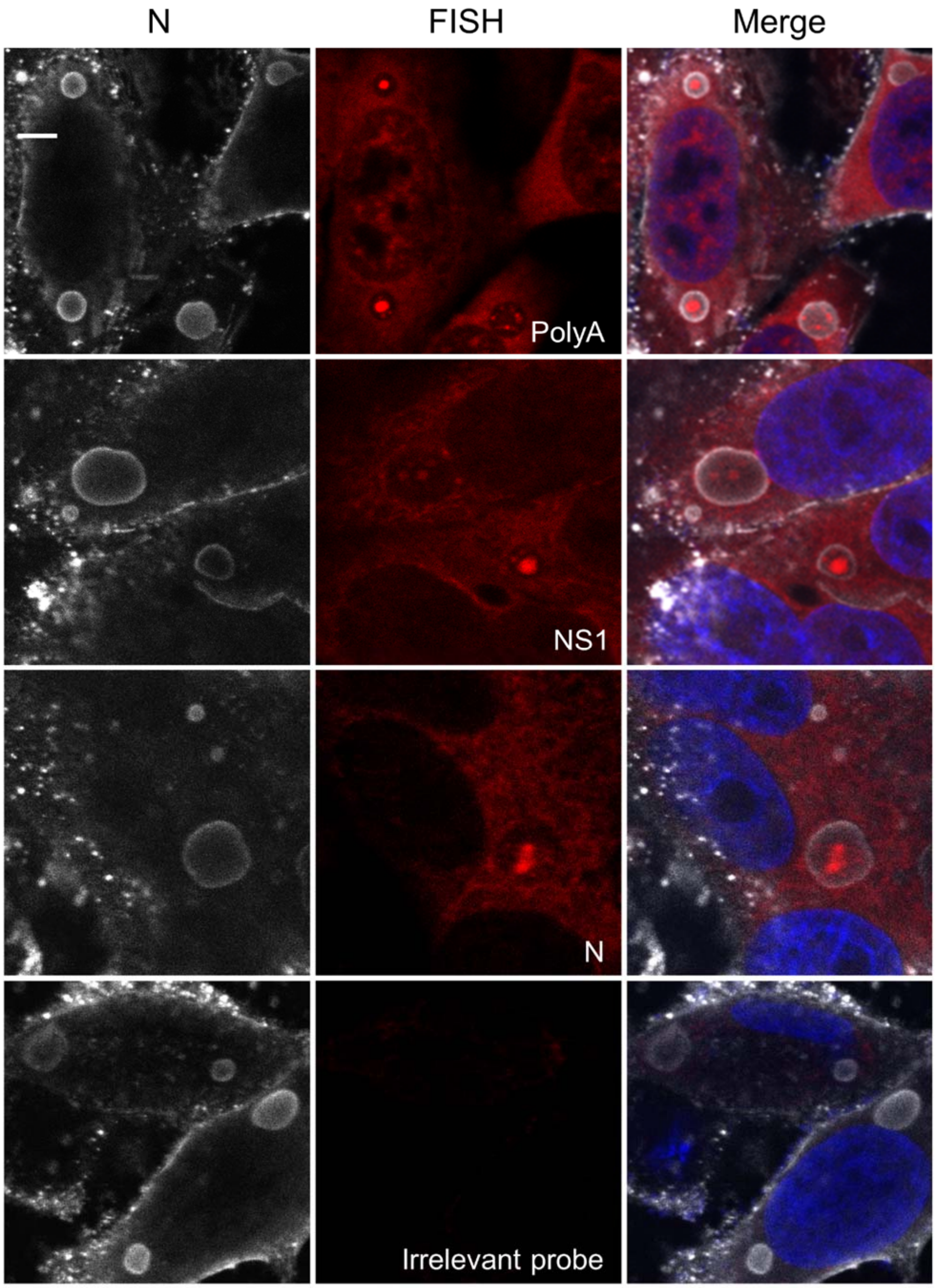

Supplementary Figure 2: IBAGs are seen in IBs of cells infected with a RSV isolated from a clinical specimen. HEp-2 cells were infected with a RSV isolated from a clinical specimen. At 24h p.i., FISH analyses were performed with specific probes (red) to detect polyadenylated RNA (PolyA), NS1 mRNA, N mRNA or the M mRNA of VSV (irrelevant probe) as indicated on the pictures. Cells were stained with an anti-N antibody (grey) and Hoechst 33258 (merge). Representative images from 3 independent experiments are shown. Images were taken under a Leica SP8 confocal microscope. Scale bar 5 µm;

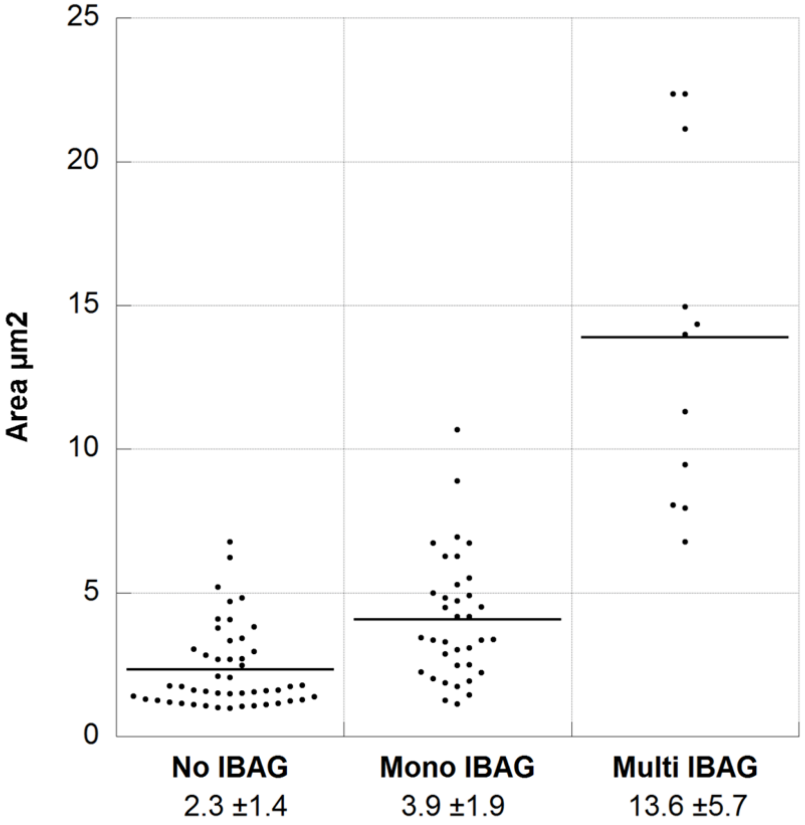

Supplementary Figure 3: Correlation between size of IBs and IBAGs detection. HEp-2 cells were infected with RSV for 24h. FISH analyses were performed with specific probes to detect polyadenylated RNA (PolyA) and stained with an anti-N antibody. Quantitative analyses were performed with ImageJ on 8 randomly selected fields. The cross-sectional areas of inclusion bodies at different time points were measured and the number of IBAGs defined by polyA spots was determined. Each plot depicts one IB, presented according to its area and category. The mean areas in μm^2 ± s.d. are indicated for each category. Bars indicate the mean areas of IBs of the different categories, which are significantly different ($p<0.001$ one way ANOVA. Normality of the distribution of the IBs size in each group was verified using Shapiro-Wilk normality test).

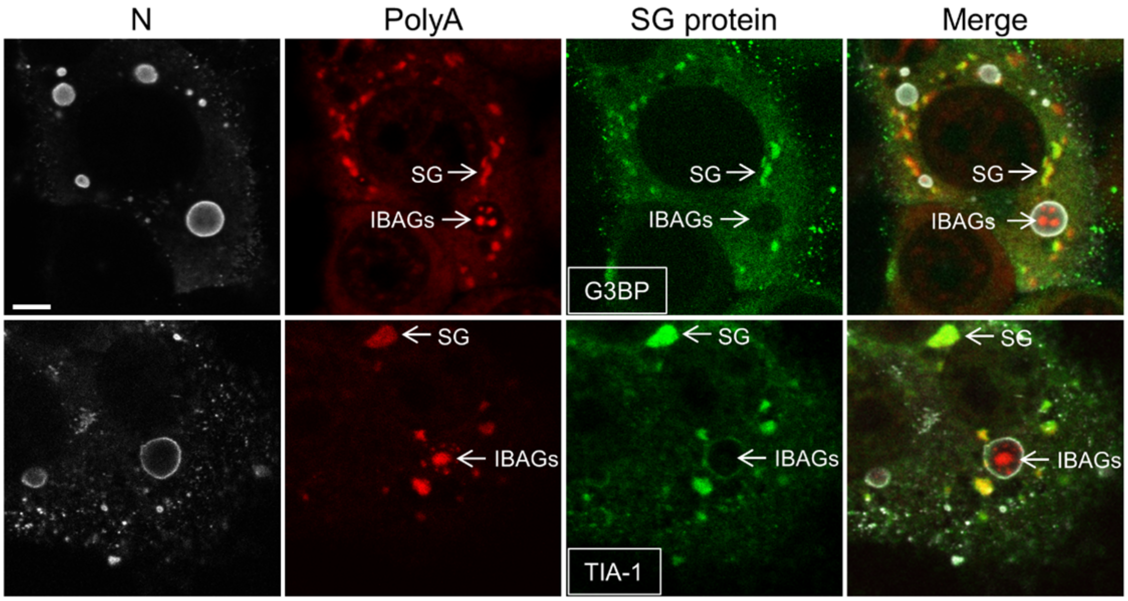

Supplementary Figure 4: IBAGs are distinct from cellular stress granules. HEp-2 cells were infected with RSV. At 24h p.i., FISH analyses were performed to detect polyadenylated RNAs (red) and cells were stained with an anti-N antibody (grey) and with anti-G3BP or anti-TIA-1 (SG proteins in green). Cells with both stress granules (SG) and IBAGs are presented. Both structures are indicated by white arrows. Images of cells exhibiting SGs were taken under a Leica SP8 confocal microscope in 3 independent experiments. Scale bar 5 µm.

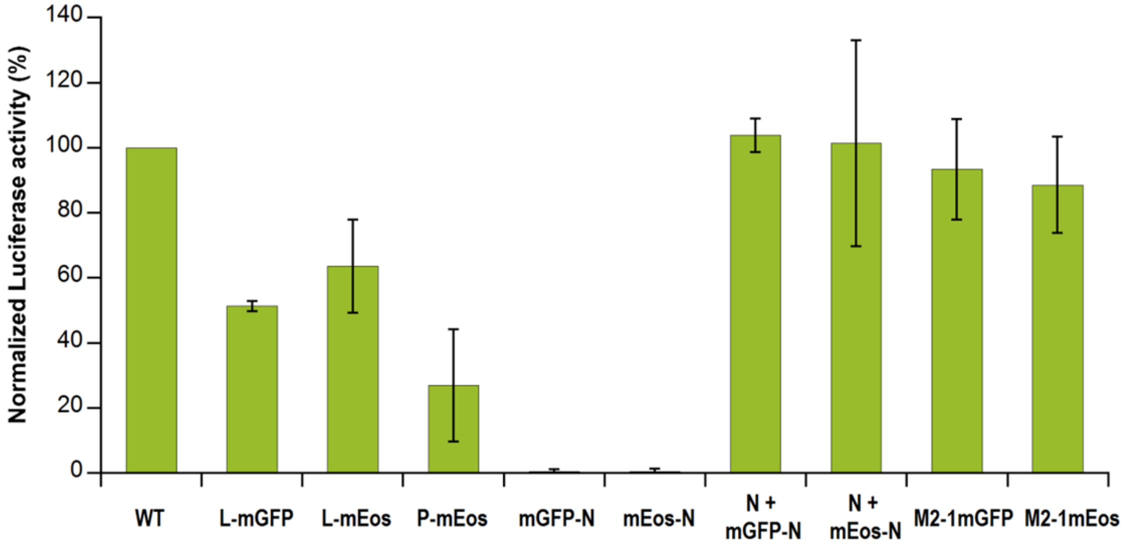

Supplementary Figure 5: Ability of tagged proteins to support RSV transcription-replication. BSRT7/5 cells were transfected with plasmids encoding the N, P, L and M2-1 proteins and the M/Luc subgenomic minireplicon together with pCMV β-gal for transfection standardization. Tagged protein was expressed instead of the corresponding wild type as indicated below the histogram. For N protein, the plasmids encoding wild type and tagged protein were mixed in equivalent ratio when indicated N + tag-N. Transcription-replication activities of the tagged proteins were characterized in the minireplicon assay. Luciferase activity, measured 24 h after transfection, was normalized to the β-galactosidase activity, and expressed as percentage of the WT proteins activity. The mean value ± standard deviations (s.d.) result from 3 to 4 independent experiments performed in triplicate.

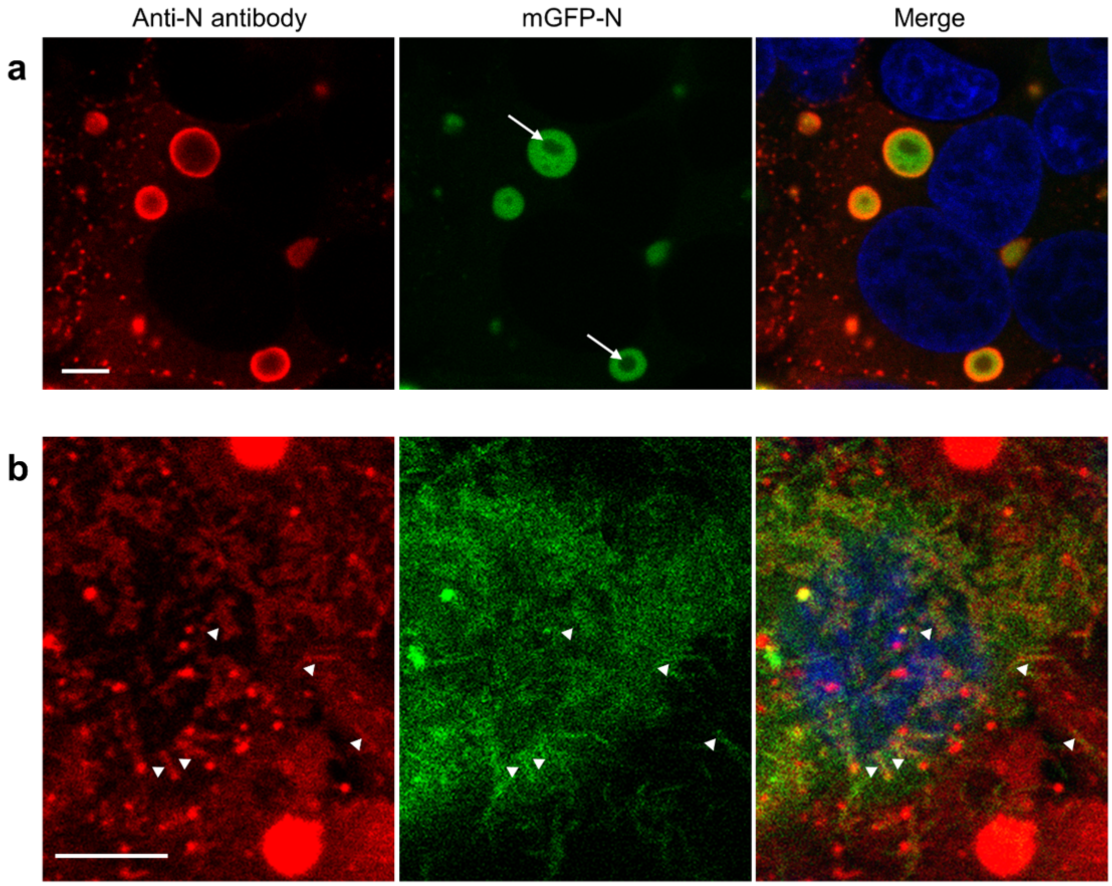

Supplementary Figure 6: Localization of mGFP-N and wild type N in RSV infected cells. BSRT7/5 cells were transfected with plasmid encoding the mGFP-N protein then infected with RSV. At 24h p.i. cells were stained with an anti-N antibody (red) and Hoechst 33258 (merge). The mGFP-N protein is visualized through its spontaneous green fluorescence. IBs are shown in (a), IBAGs are indicated with white arrows. Viral filaments are shown in (b) and indicated with white arrowheads. Representative images from 2 independent experiments are shown. Images were taken under a Leica SP8 confocal microscope. Scale bars 5 µm.

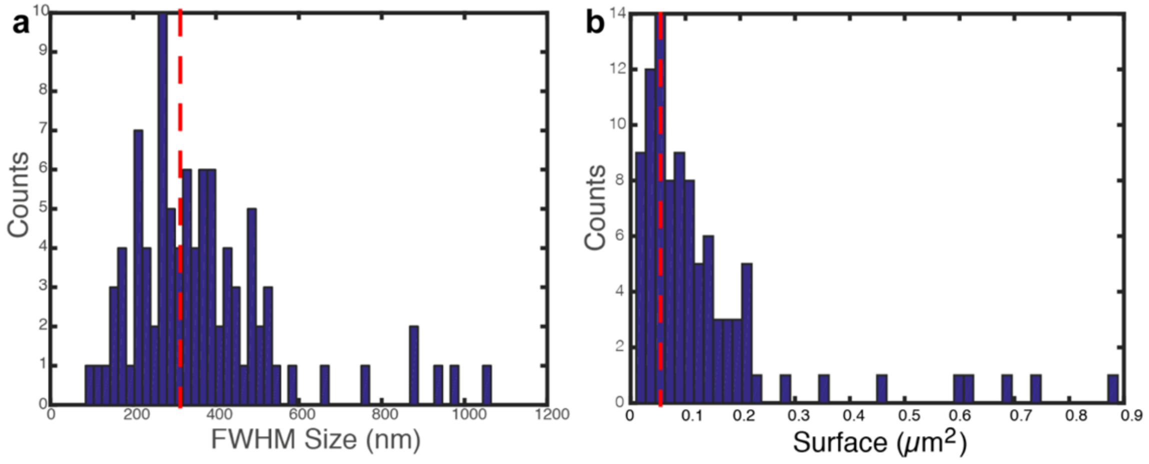

Supplementary Figure 7: Size distribution of IBAGs from super-resolution images. Histograms of the sizes (**a)** and surfaces (**b**) of poly-A clusters. For these histograms, 100 high-resolution clusters of poly-A were manually selected in the PALM/STORM image. For each cluster, we measured σ_x and σ_y as the standard deviation of the *x* and *y* coordinates of poly-A localizations, respectively, and computed the full width at half maximum (FWHM) size as $2.355\sqrt{\sigma_x\sigma_y}$ and the surface as $\pi\sigma_x\sigma_y$. Red lines indicate the diffraction limit to resolution (~300nm) for Alexa647 (with λ 700nm and numerical aperture NA=1.45).

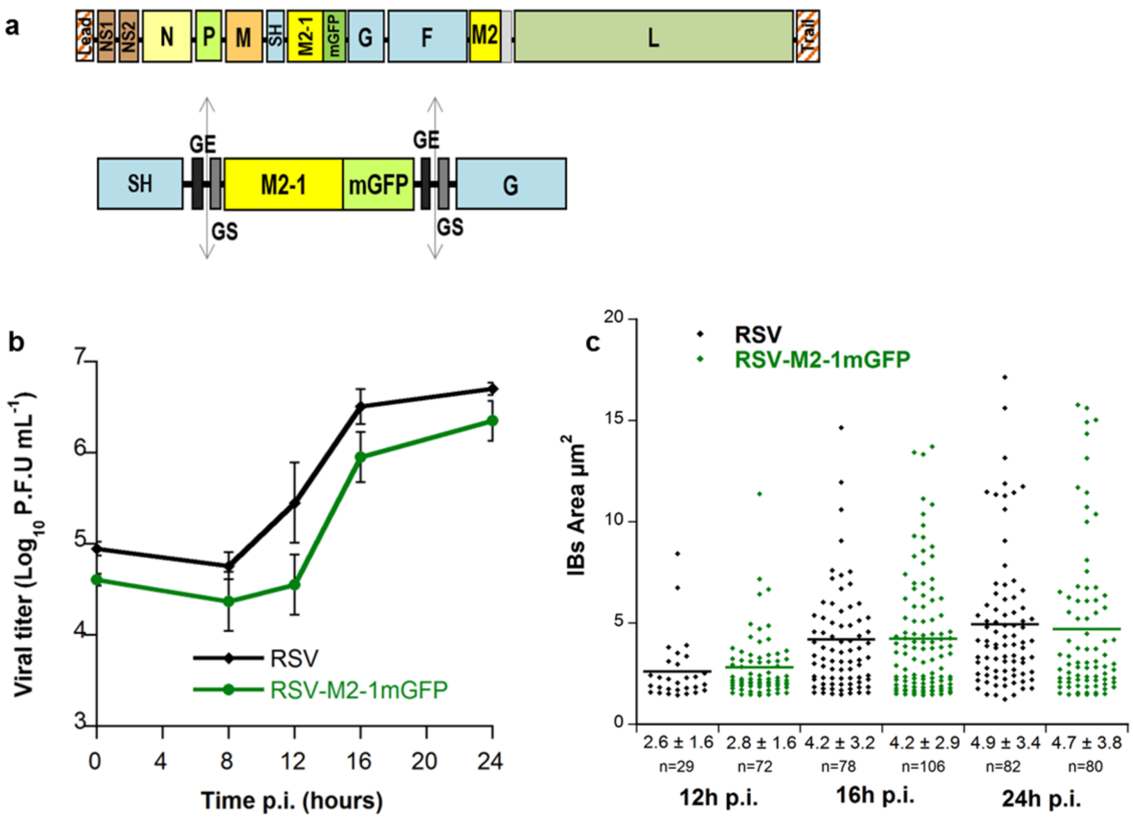

Supplementary Figure 8: (A) Schematic representation of RSV-M2-1mGFP infectious clone (not to scale). Protein-encoding frames are shown as colored boxes, leader and trailer regions as hatched boxes. Intergenic regions are shown as a black horizontal line. The M2-1mGFP coding sequence was inserted between SH and G genes together with an upstream gene end (GE) and a downstream gene start (GS) signals as described in Methods. **(B) Growth properties of RSV-M2-1mGFP**. HEp-2 cells were infected with the RSV-M2-1mGFP or RSV at a MOI of 1 at 37°C and viruses were harvested at the indicated times p.i. and titrated by plaque assays on HEp-2 cells. Results are the mean ± s.d. for three independent experiments. Titers of RSV and RSV-M2-1mGFP at the different time points are not significantly different except for the t0 (t test values on logarithmic-transformed data t0:4.13; t8:1.34; t12:1.99; t16:2.03; t24:1.88, 4 degrees of freedom, variances of compared groups are not statically different). **(C) Time course analysis of IBs formation in RSV and RSv-M2-1mGFP infected cells.** HEp-2 cells were infected with RSV and RSV-M2-1mGFP. Cells were also stained with an anti-N antibody and Hoechst 33258 at the indicated time p.i.. Quantitative analyses were performed with ImageJ on 3 randomly selected fields (about 40 cells). The cross-sectional areas of inclusion bodies at different time points were measured. Each plot depicts one IB, presented according to its area. The mean areas in μm^2 ± s.d. and the number of IBs are indicated for each category.

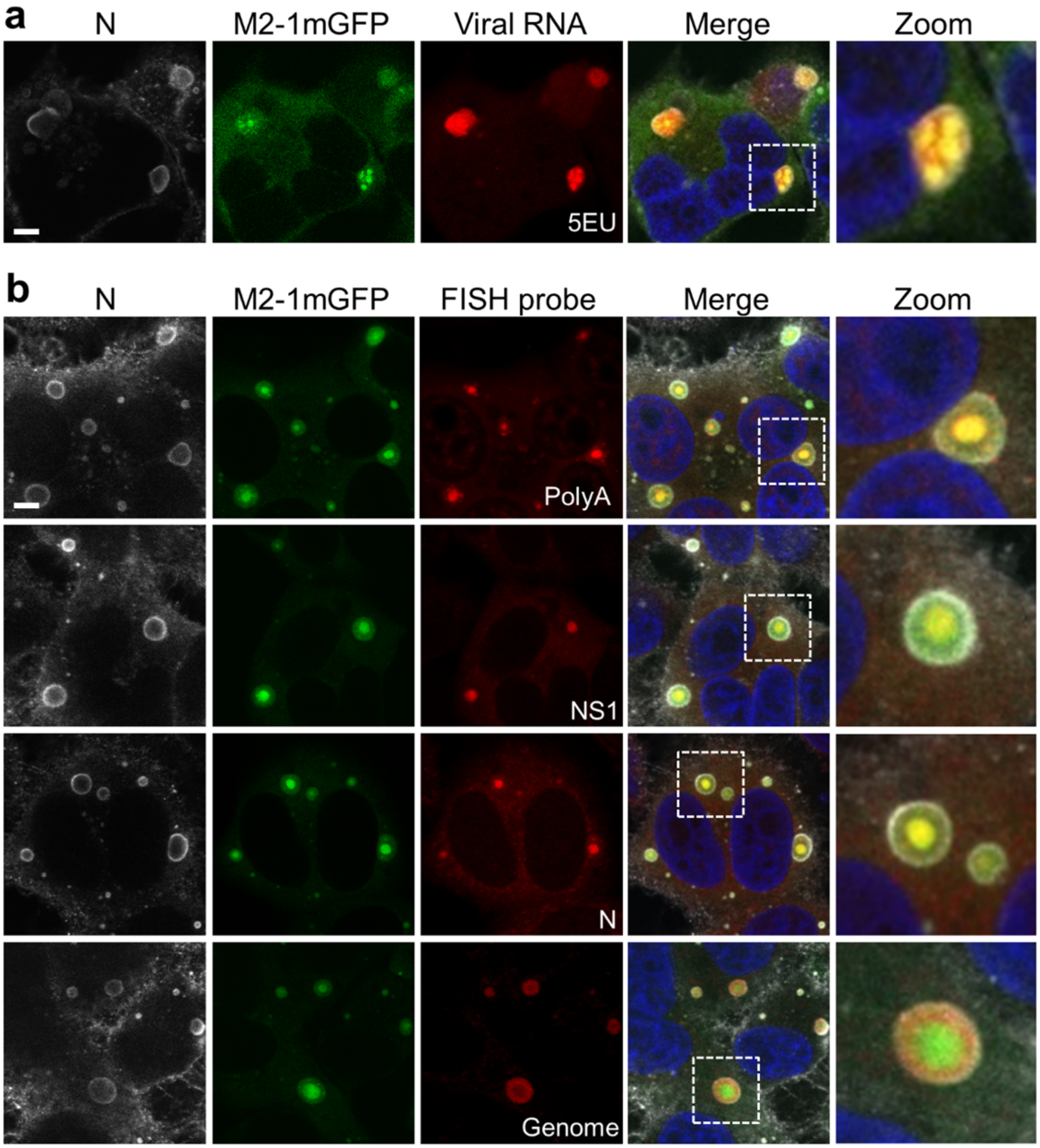

Supplementary Figure 9: M2-1mGFP concentrates in IBAGs with newly synthetized viral mRNA in RSV-M2-1mGFP infected cells. (a) HEp-2 cells were infected with RSV-M2-1mGFP. At 24h p.i. cells were incubated for 1h with actinomycin D to inhibit cellular transcription and then with 5-ethynyl uridine (5EU) for one more hour before cell fixation. The 5EU incorporated in newly synthesized RNAs was detected using Alexa Fluor 647-azide (red). **(b)** At 24h p.i., FISH analyses were performed with specific probes (red) to detect polyadenylated RNA (PolyA), NS1 mRNA, N mRNA, viral genomic RNA or the M mRNA of VSV (irrelevant probe) as indicated on the pictures. Cells were stained with an anti-N antibody (grey) and Hoechst 33258 (merge) (**a**) and (**b**). The M2-1mGFP protein is visualized through its spontaneous green fluorescence. Representative images from 4 independent experiments are shown. Images were taken under a Leica SP8 confocal microscope. The boxed areas enclose IBs that are shown magnified (zoom). Scale bars 5 µm.

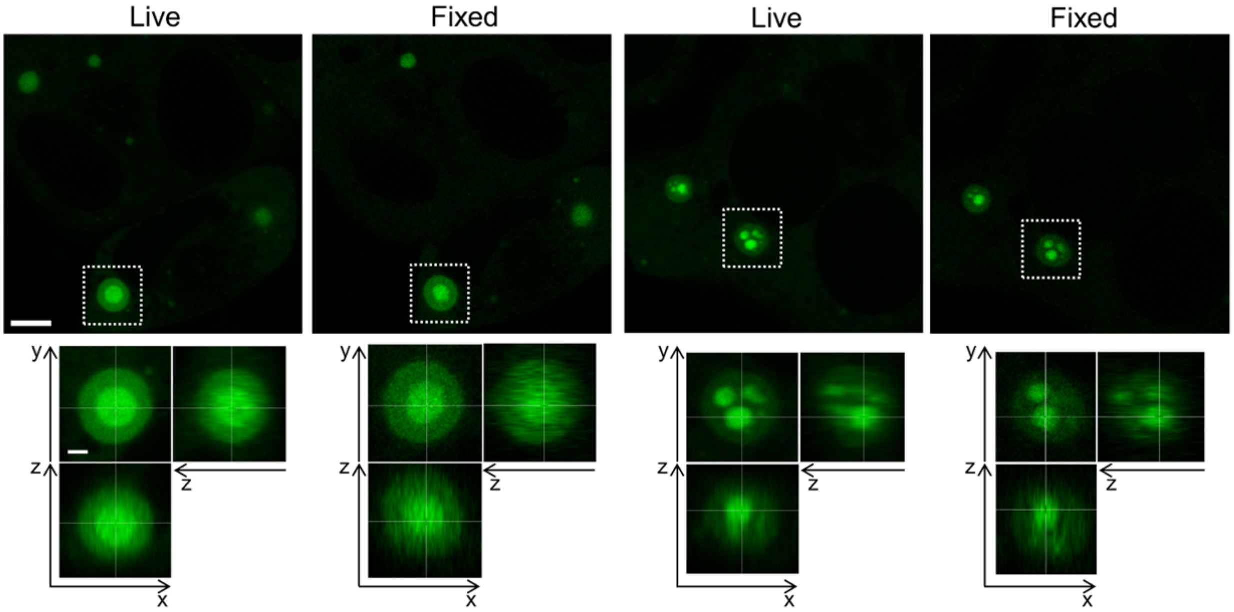

Supplementary Figure 10: Comparison of IBs morphology in fixed and living cells

HEp-2 cells were infected with RSV –M2-1mGFP for 24h. Images were taken with living cells just before fixation. Images of the same cells were taken after fixation. Representative images are shown. Images were taken under a confocal microscope, scale bar 5µm. The boxed areas enclose IBs that are shown magnified (zoom), scale bar 1µm. Single x-y, x-z and y-z sections are shown as indicated. The dynamic behavior of IBAGs, and IBs to a lesser extent, explains the slight differences observed between living and fixed cells.

Supplementary table 1: Sequences of the primers used to construct the pACNR-rHRSV-M2-1mGFP

Primer	Sequence 5'-3'
M2-1mGFP for	AAAAAGGGTAACCTGGGGCAAATATGGCACGAAGGAATCCTTGCAAATTTG
M2-1mGFP rev	AATAAAGGTTACCATGTGTATAATTTTTAATTAACTCATTTGTACAGCTCGTCCATGCCGAGAG
